# Explore the Value of Dual Source Computer Tomography Automatic Tube Current Regulation in Reducing the Radiation Dose of CTA in Lower Extremity Vessels

**DOI:** 10.3389/fsurg.2022.896370

**Published:** 2022-05-03

**Authors:** Xin Hu, Yi Yu

**Affiliations:** ^1^ Department of Guangdong Armed Police Corps Hospital, Medical Engineering, Guangzhou, Guangdong, China; ^2^ School of Foreign Languages of Guangdong University of Technology, Guangzhou, Guangdong, China

**Keywords:** dual source computer tomography, automatic tube current regulation technology, CTA, radiation dosage, extremity vessels

## Abstract

**Objective:**

To investigate the value of dual source computer tomography automatic tube current regulation in reducing the radiation dose of CTA in lower limb vessels.

**Methods:**

From February 2020 to December 2021, 64 patients with lower limb artery CTA were selected in our hospital because of the symptoms of foot ischemia. According to the random number table, patients were divided into control group (treated with fixed tube current technology) and observation group (treated with automatic tube current regulation technology), with 32 cases in each group. All patients underwent a dual source computer tomography scan. Control group: tube voltage 120 kV, tube current 250 mA; Observation group: tube voltage was 80 kV, and reference tube current was 80–380 mA. Other scanning conditions of patients in the two groups were the same. CTDIvol, DLP and calculated SNR and CNR were recorded to obtain the ED.

**Results:**

The values of CTDIvol, DLP and ED in the observation group were lower than those in the control group (*P *< 0.05). There was no significant difference in CT value, SD value, SNR value and CNR value of the femoral artery segment, popliteal artery segment and posterior tibial artery segment between the two groups (*P *> 0.05). The image quality scores of patients in the control group were slightly higher than those in the observation group, but there was no statistical difference between the two groups (*P *> 0.05).

**Conclusion:**

The application of dual source computer tomography automatic tube current adjustment technology in CTA examination of lower limb vessels can automatically adjust the compensation output and realize the output of different tube currents in different thicknesses, densities and angles. On the premise of not affecting the image quality, the radiation dose in the scanning process to the maximum extent, and reasonably protect the examined patients.

## Introduction

Arterial disease of the lower limbs is a kind of vascular disease that seriously harms human health, and it involves a wide range of asperts. In the later stage, it is mainly manifested as severe stenosis or occlusion of blood vessels, aneurysm formation, etc., which easily leads to ischemia or necrosis of the distal limbs, and often requires surgery or intervention therapy (1, 2). At present, the examination of lower limb arterial obstructive disease mainly diagnoses the cause, location, scope and degree of the disease when the disease occurs in the main artery or larger branch. Imaging examination is an important basis and prerequisite for identifying lesions and making treatment plans (3, 4). Although digital subtraction angiography is the gold standard of blood vessel examination, it has not been widely accepted as a routine blood vessel examination because of its invasiveness, high cost, and many complications.

With the rapid development of CT, the imaging speed of CT angiography is fast, relatively cheap, and there are few complications. Clinically, it is favored by doctors and patients, and it is a common means to diagnose vascular diseases at present. However, with its increasing clinical applications, the radiation dose has attracted more and more attention (5, 6). In many developed countries, CT is regarded as the most important cause of iatrogenic radiation. A wide range CTA scan of lower limb blood vessels using routine scanning will lead to an increase in the radiation dose of the subjects and the potential incidence of cancer in the exposed population (7). CTA scanning range of lower limbs is long, and there are obvious differences in thickness and density of each segment. If the current and voltage of the tube are fixed too high during the whole process, it will cause unnecessary radiation, or too low voltage will affect the image quality (8). Therefore, how to optimize the combination of tube voltage and tube current to reduce the scanning radiation dose and reduce the potential radiation damage is of paramount importance. In this study, the effective tube current was calculated based on the ray absorption by different parts of the human body and the shape of the scanned object to automatically adjust, and whether the automatic tube current adjustment technology could achieve the purpose of reducing the radiation dose while ensuring the image quality was discussed.

## Data and Methods

### General Information

From February 2020 to December 2021, 64 patients with lower limb artery CTA were selected in our hospital because of the symptoms of foot ischemia. Inclusion criteria: Age ≥18 years old; BMI 20–30 kg/m^2^; The patient cooperated well. Exclusion criteria: Allergy to iodine contrast agent; Severe cardiac and renal insufficiency; Hyperthyroidism; Pregnant or lactating women. The patients were divided into a control group and an observation group according to the random number table, with 32 cases in each group. The control group was treated with fixed tube current technology, while the observation group was treated with automatic tube current regulation technology. In the control group, there were 22 males and 10 females, with the average age of (54.81 ± 7.94) years old and body mass index (BMI) (24.84 ± 1.46) kg/m^2^. In the observation group, there were 24 males and 8 females, with the average age of (55.17 ± 8.01) years old and BMI (24.73 ± 1.39) kg/m^2^. This study was approved by the Hospital Ethics Committee and all patients signed informed consent forms before the examination.

### Research Methods

#### Inspection Methods

All patients underwent a dual source computer tomography scan (SOMATOM Definition CT, DSCT). Scanning range: From the level of the third lumbar vertebra (including part of the abdominal aorta) to the foot end, to both soles of the feet. Supine position foot advanced, calm breathing. 120 mL of iopamidol (370 mgI/mL) was used as the contrast agent, and the injection was conducted in two consecutive times. The first injection was conducted with 80 mL, and the flow rate was 4.0 mL/s; A second injection of 40 mL was performed at a flow rate of 3.0 mL/s. After the injection of contrast agent, 50 mL normal saline was injected with a flow rate of 4.0 mL/s. A region of interest was selected at the popliteal fossa for monitoring using contrast tracking techniques. The trigger level was distal to the abdominal aorta, and the scan was manually triggered after the CT value reached the preset value (100 HU). Other scanning conditions of the patients in the two groups were the same: reconstruction layer thickness of 0.75 mm, reconstruction interval of 0.5 mm, pitch of 1.375, collimation of 64 mm × 0.6 mm, reconstruction matrix of 512 × 512, and reconstructed field of view of 185–200 mm. Control group: tube voltage 120 kV, tube current 250 mA; Observation group: tube voltage was 80 kV, and reference tube current was 80–380 mA.

#### Radiation Dose Assessment

The scan length after examination for each patient was calculated. The CT volume dose index (CTDIvol) and the dose length product (DLP) automatically calculated by the device were recorded in the image data column after the scan to obtain the effective dose (ED)  = DLP × W (W = 0.019 mSv·mGy^−1^·cm^−1^) (9).

#### Image Evaluation

The scanned data were transmitted to the workstation for volume rendering, multiplanar reconstruction, maximum intensity projection and curved surface reconstruction, followed by post-processing of the images.

The subjective 3-segment scoring was performed by two senior physicians in the cardiovascular group using a double-blind method. First segment: from the distal end of the abdominal aorta to the branch of the internal and external iliac arteries of the common iliac artery; Second segment: from the branch of internal and external iliac artery of the common iliac artery to the anterior and posterior tibial artery of the popliteal artery; Third segment: The popliteal artery runs from the anterior and posterior tibial arteries to the following parts. According to the main points of lower extremity artery diagnosis, the scoring criteria were established as follows: 3 points: The lumens of arteries and their branches in the 3 groups were well filled with contrast agent, which could clearly show the calcification of the vessel wall, the degree and extent of stenosis, and the branches of arterioles. 2 points: The lumens of arteries and their branches in the three groups were better filled with contrast agent, which could better show the calcification of the vascular wall, and the degree and scope of stenosis. The branches of arterioles showed better. 1 points: The arteries and their branches in the three groups showed poor contrast, the vessel wall was rough, and the branches and segments of some arterioles were poor. 0 points: Only the morphology of the main blood vessels in the three groups could be displayed, and the distal vessels and branches were not fully displayed or could not be displayed.

Calculating a signal-to-noise ratio (SNR), wherein SNR = arterial CT enhanced scan value/image noise SD value; Contrast signal-to-noise ratio (CNR), CNR = (arterial CT enhancement scan value-muscle tissue CT value)/image noise SD value. Measure that CT values of the midpoint of the common iliac artery, the midpoint of the external iliac artery and the midpoint of the femoral artery and calculate the average CT value representing a target tissue signal; The standard deviation (SD) of the ambient air CT value measured at the midpoint of the bilateral common iliac arteries represented image noise; The measure mean CT values of that bilateral psoas muscle represent background signals.

### Statistical Methods

SPSS22.0 software was used for processing. Measurement data such as CTDIvol, DLP, and ED values of the experimental data were expressed as mean ± standard deviation $(\bar{x}\pm s)$, and pairwise comparison of measurement data between groups was analyzed by *t* test. The test level was α = 0.05, and *P *< 0.05 indicated that the difference was statistically significant.

## Results

### Comparison of Radiation Doses Between the Two Goups

**Figure 1:**
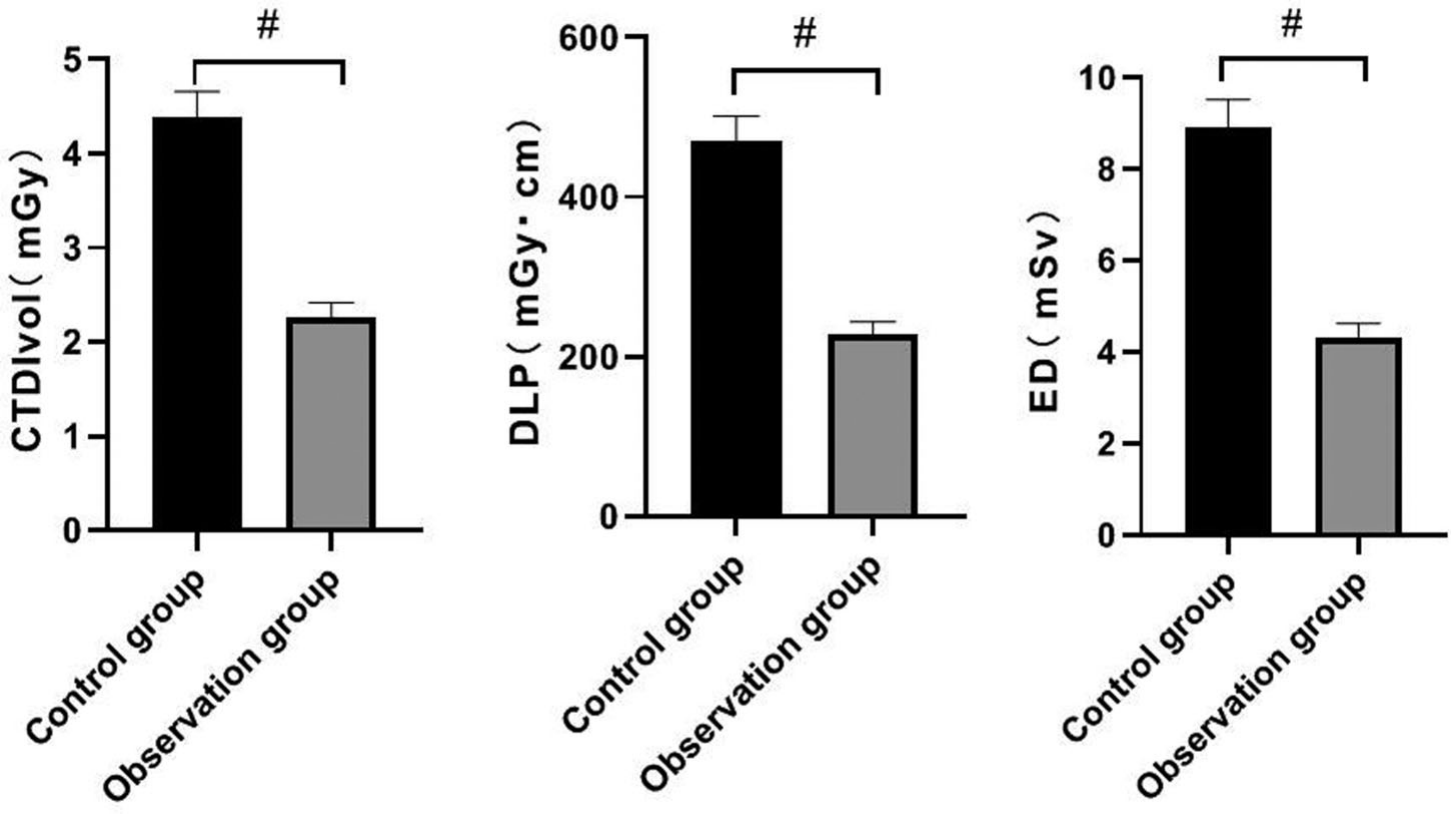
Comparison of radiation doses between the two groups. Note: Compared with the control group, ^#^*P *< 0.05.

The values of CTDIvol, DLP and ED in the observation group were lower than those in the control group (*P *< 0.05). See **Figure 1**.

### Comparison of Measured Values of Femoral Artery Segment Between the Two Groups

**Figure 2:**
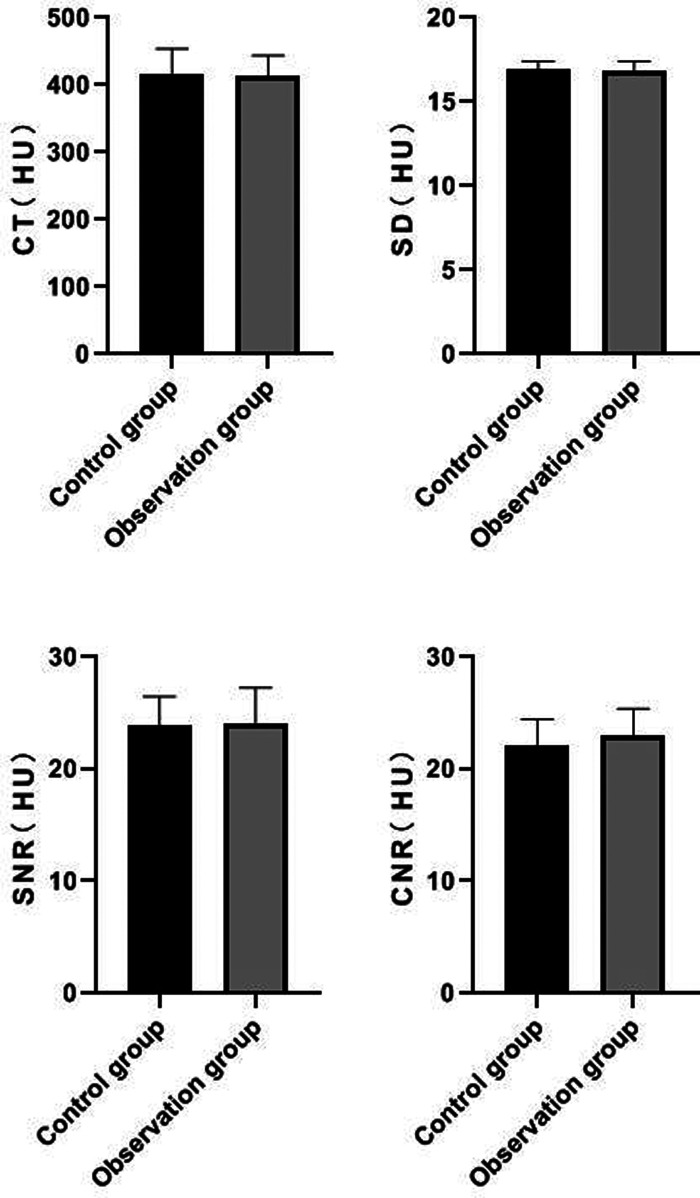
Comparison of measured values of femoral artery segment between the two groups.

There was no significant difference in CT value, SD value, SNR value and CNR value of femoral artery segment between the two groups (*P *> 0.05). See **Figure 2**.

### Comparison of Measured Values of Popliteal Artery Segment Between the Two Groups

**Figure 3:**
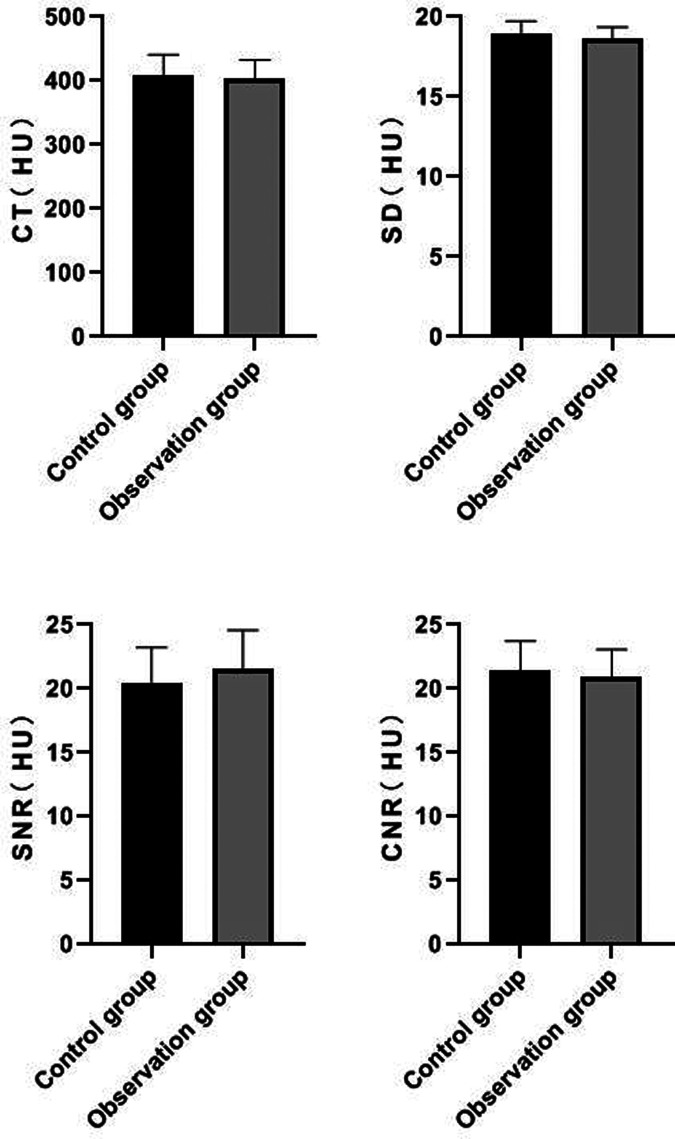
Comparison of measured values of popliteal artery segment between the two groups.

There was no significant difference in CT value, SD value, SNR value and CNR value of popliteal artery segment between the two groups (*P *> 0.05). See **Figure 3**.

### Comparison of Measured Values of Posterior Tibial Artery Segment Between the Two Groups

**Figure 4:**
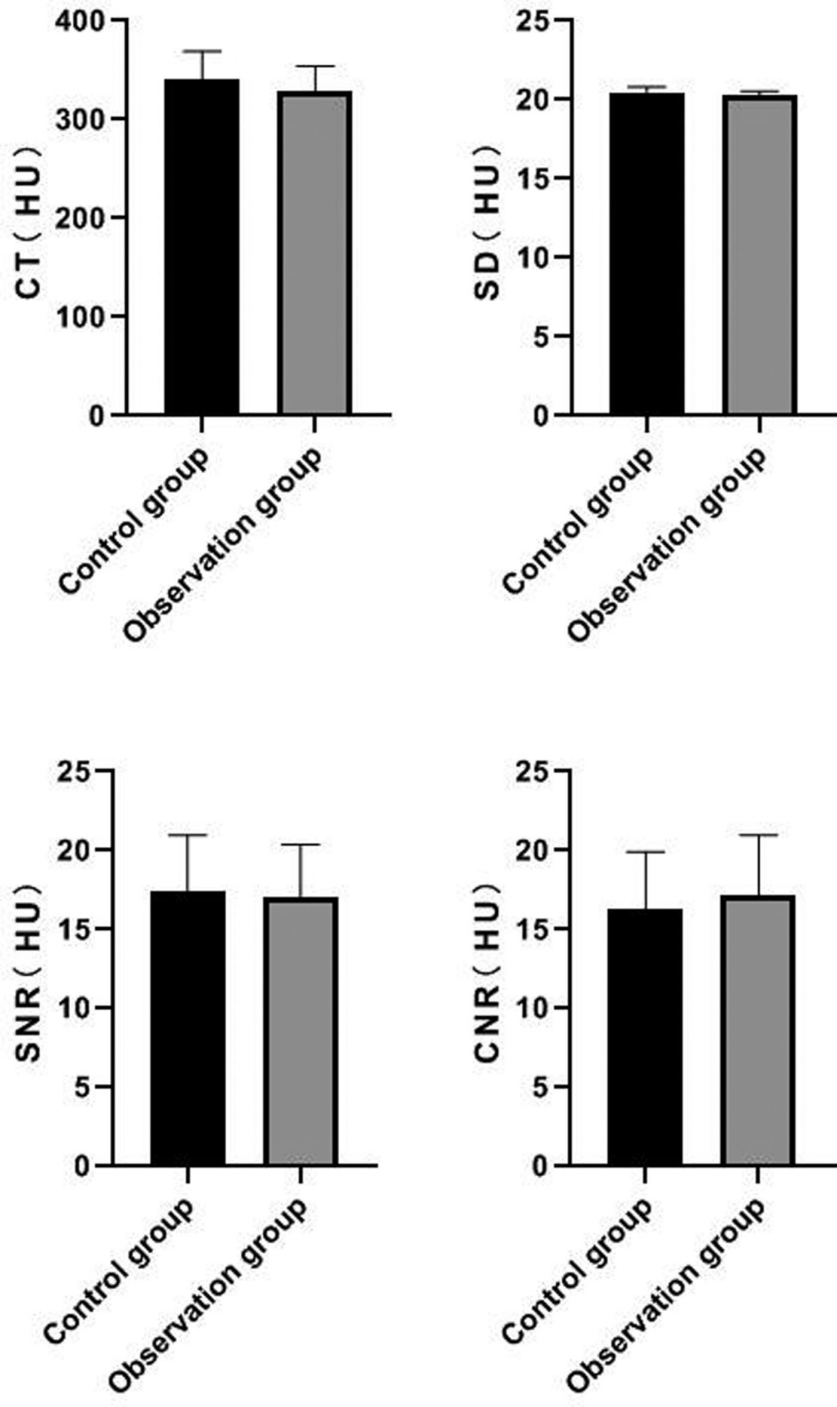
Comparison of measured values of posterior tibial artery segment between the two groups.

There was no significant difference in CT value, SD value, SNR value and CNR value of posterior tibial artery between the two groups (*P *> 0.05). See **Figure 4**.

### Comparison of Image Quality Scores Between the Two Groups

**Figure 5:**
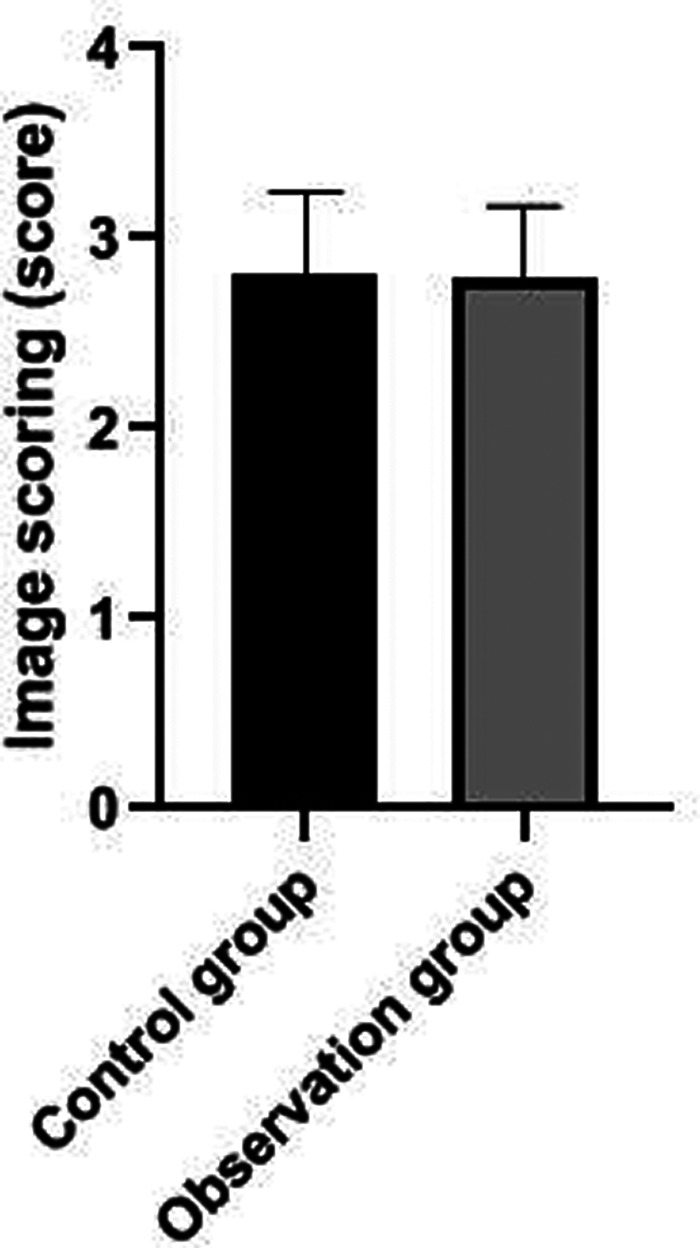
Comparison of image quality scores between the two groups.

The image quality scores of patients in the control group were slightly higher than those in the observation group, but there was no statistical difference between the two groups (*P *> 0.05). See **Figure 5**.

## Discussion

The harm of X-ray radiation used in medical diagnosis is mainly carcinogenic and teratogenic, and the harm degree is related to the absorbed dose. According to the estimation of the “Report of the Committee on the Biological Effects of Ionizing Radiation VII”, for every 10,000 population in the United States, 1 case of radiation-induced cancer is added for every 1 mSv exposure (10). With the development of CT, the frequency of examination has doubled all over the world, and the radiation dose generated from CT examination accounts for about half of the total medical radiation dose. CT radiation dose is related to tube voltage, tube current, pitch, scanning range and collimator width. Other factors related to radiation dose are also closely related to image quality. Therefore, how to ensure the image quality while minimizing the radiation dose has become one of the research directions of CT (11–13).

In the CTA examination of lower limb blood vessels, the CTA scanning range of the lower limb is long. After passing through the gonadal organs (radiation-sensitive organs), the thicknesses of tissues in each segment is quite different (such as the pelvic cavity, knee joint and ankle joint), and the distribution range of tissues in the whole segment is uneven (14, 15). Because the tube current needs to be multiplied by the downcomer voltage to ensure the image quality, if the tube voltage is greatly reduced and the tube current is doubled, the radiation dose may not be reduced, and the image quality may suffer (16–18). Therefore, in the process of reducing the radiation dose, it is difficult to grasp the adjustment of various parameters, and it is impossible to give considerations to the image quality and radiation dose. If we want to ensure the image quality and reduce the radiation dose, we must choose the best combination of tube voltage and current (19–21).

In this study, the automatic tube current regulation technology was used to make the tube current float up and down within a certain range. When the tube voltage at the thicker part was insufficient, the tube current is compensated to ensure the image quality. For the thinner part, the output of tube current is automatically reduced to reduce radiation dose, and the optimal combination of tube voltage and tube current can be realized according to different thicknesses and densities. The results showed that the values of CTDIvol, DLP and ED in the observation group were lower than those in the control group. It shows that the application of automatic tube current adjustment technology in CTA of lower limb, make accurate assessment of lower limb artery stenosis, display the location and degree of the lession from multiple angles and directions, and reduce the radiation dose on the premise of ensuring the image quality.

Applying the technique of automatic tube current adjustment, appropriately increase the tube current of iliac artery and appropriately decreasing the tube current of thigh and calf artery can ensure the noise consistency of the whole lower limb scanning range and reduce unnecessary radiation dose (22–24). The results of this study showed that there was no significant differences in CT, SD, SNR, and CNR values of femoral artery, popliteal artery and posterior tibial artery between the two groups. It could be seen that the automatic tube current regulation technology had little influence on the SD value of high-density tissue while ensuring the good filling of the contrast media in the vascular lumen. The results also showed that the image quality scores of the control group were slightly higher than those of patients in the observation group, but there was no statistical difference between the two groups. According to the international radiological examination, we should follow the principle of “rational use of low dose”, carefully select scanning parameters, optimize all scanning procedures, and allow certain noise in according to different clinical requirements on the premise of meeting the requirementa of clinical diagnosis, so as to reduce the radiation dose as much as possible (25, 26). CTA of lower limb vessels mainly observes the arterial vessels, but does not require high image quality of other tissues. The filled high-density blood vessels and surrounding tissues have high CNR, and the whole segment and important branches of the blood vessels can be clearly displayed through various post-processing methods, so there is no significant difference in quality score results of reconstructed blood vessel images (27, 28).

Limitations of this study: ① The radiation dose recorded by two groups of patients with fixed tube current and automatic tube current adjustmengt is not as accurate as that recoeded by the same group of patients with two different examination methods. ② The data of patients with BMI within the range of 20–30 kg/m^2^ were only collected in the study, while patients with other BMI ranges were not included in the study. The cumulative number of case in follow-up research is needed to further support this conclusion.

In summary, the application of dual source computer tomography automatic tube current adjustment technology in CTA examination of lower limb vessels can automatically adjust the compensation output and realize the output of different tube currents in different thicknesses, densities and angles. On the premise of not affecting the image quality, the radiation dose in the scanning process to the maximum extent, and reasonably protect the examined patients.

## Data Availability

The original contributions presented in the study are included in the article/supplementary material, further inquiries can be directed to the corresponding author/s.
